# The impact of sodium glucose cotransporter-2 inhibitors on ventricular arrhythmia in patients with implantable cardioverter-defibrillators: a meta analysis

**DOI:** 10.1007/s10840-026-02274-1

**Published:** 2026-02-28

**Authors:** Mayank Jha, Danielle Serafim Howell, Oybek Nazarov, Kruti Matawala, Deep Chahodiya, David Kim, Mohamed Ali, Jubran Al Hooti, Hritvik Jain, David Song, Seth Goldbarg

**Affiliations:** 1 Department of Internal Medicine, Government Medical College and New Civil Hospital, Surat, India; 2 Department of Internal Medicine, New York Presbyterian Queens, Weill Cornell Medicine, Flushing, NY USA; 3 Department of Internal Medicine, Touro University, New York City, USA; 4 Department of Internal Medicine, University College Dublin, Dublin, Ireland; 5 Department of Internal Medicine, All India Institute of Medical Sciences, Jodhpur, India; 6 Department of Cardiovascular Disease, New York Presbyterian Queens, Weill Cornell Medicine, Flushing, NY USA; 7 Department of Electrophysiology, New York Presbyterian Queens, Weill Cornell Medicine, Flushing, NY USA

**Keywords:** Sodium-glucose cotransporter 2 inhibitors, Implantable cardioverter debrillator, Arrhythmias, Ventricular Tachycardia, Cardiovascular Mortality

## Abstract

**Introduction:**

Sodium-glucose cotransporter-2 (SGLT2) inhibitors improve outcomes in heart failure (HF), but their effect on arrhythmias is less clear. Patients with implantable cardioverter-defibrillators (ICDs) are a high risk population for ventricular arrhythmias. However, the effects of SGLT2 inhibitors on ventricular arrhythmias remains a source of debate.

**Methods:**

We systematically searched PubMed, Scopus, and Cochrane databases from inception to August 19, 2025, for studies evaluating SGLT2 inhibitors and arrhythmic/cardiovascular outcomes. Pooled mean differences (MD) and risk ratios (RR) with 95% confidence intervals (CI) were calculated using a random-effects model.

**Results:**

A total of 1,605 patients were included in our analysis. SGLT2 inhibitors was associated with a significant reduction in the risk of ventricular arrhythmia (RR: 0.38; 95% CI: 0.17 to 0.81; *p* = 0.01), ventricular tachycardia (RR: 0.60; 95% CI: 0.41 to 0.88; *p* = 0.009), and supraventricular tachycardia (RR: 0.67; 95% CI: 0.47 to 0.95; *p* = 0.02). No significant effect was observed on non-sustained VT (RR: 0.94; 95% CI: 0.52 to 1.67; *p* = 0.83). However, SGLT2 inhibitors were also associated with a significant reduction in ICD shocks (RR: 0.49; 95% CI: 0.38 to 0.64; *p* < 0.00001) and cardiovascular mortality (RR: 0.39; 95% CI: 0.22 to 0.70; *p* = 0.002).

**Conclusion:**

In ICD patients, SGLT2 inhibitor therapy significantly reduced ventricular arrhythmias, discrete sustained VT, and SVT, with secondary analyses suggesting fewer ICD shocks and reduction in CV mortality in the patients with ICD.

**Supplementary Information:**

The online version contains supplementary material available at https://doi.org/10.1007/s10840-026-02274-1.

## Introduction

Sudden cardiac death from arrhythmias contributes to a significant portion of mortality in HF patients, representing a major health care burden [1]. Implantable cardioverter-defibrillators (ICDs) reduce sudden cardiac death (SCD) from ventricular arrhythmias in at-risk patients, as demonstrated in randomized trials [2]. ICDs are indicated for primary or secondary prevention, with widespread use and an estimated approximately 150,000 implants performed annually in the United States [3].

While ICD therapy may abort a malignant arrhythmia, ICD shocks are associated with increased mortality and diminished quality of life [4, 5]. HF patients are at elevated risk for atrial arrhythmias as well [6]. Several sequelae of HF appear to contribute to the development of arrhythmias, including structural heart changes and metabolic derangements. Guideline-directed medical therapy aims to target these pathologic mechanisms and has been shown to improve clinical outcomes in this patient population [7, 8].

SGLT2 inhibitors, originally developed for the treatment of type 2 diabetes, act in the renal proximal tubule to reduce glucose reabsorption. Large HF trials, including DAPA-HF and EMPEROR-Reduced, demonstrated reductions in cardiovascular death and HF hospitalization among patients with reduced LVEF, while EMPEROR-Preserved extended these benefits to patients with preserved LVEF. These agents are now widely used in HF irrespective of diabetes status [9–11]. Given the proven reductions in HF mortality and hospitalizations with SGLT2 inhibitors, their effects on arrhythmia burden have been an active area of investigation.

Evidence of antiarrhythmic effects of SGLT2 inhibitors has been mixed in prior meta-analyses. Liao et al. reported lower risks of atrial fibrillation and flutter–particularly among men and in patients with HF with reduced ejection fraction (HFrEF)-and a reduction in SCD, with the strongest effects observed for dapagliflozin [12]. Wang et al. similarly found reduced rates of atrial arrhythmias in patients with type-2 diabetes mellitus, chronic kidney disease (CKD), and HF treated with SGLT2 inhibitors. However, they observed no significant reduction in ventricular arrhythmias [13]. In contrast, Fujiki et al. and Lin et al. observed fewer ventricular arrhythmias with SGLT2 inhibitors, with Fujiki et al. attributing this benefit specifically to empagliflozin [14, 15]. These discrepancies likely reflect methodological heterogeneity, including differences in endpoints, follow-up duration, and study populations, and thus potentially limiting the power to detect effects on ventricular arrhythmias.

Given these conflicting findings, we conducted a meta-analysis to more definitively assess the impact of SGLT2 inhibitor therapy on atrial and ventricular arrhythmias among patients with ICDs.

## Methodology

We conducted a systematic review and meta-analysis in accordance with the Preferred Reporting Items for Systematic Reviews and Meta-analyses (PRISMA) statement and the Cochrane Handbook for Systematic Reviews of Interventions [16, 17]. The prespecified study protocol was registered with PROSPERO (CRD420251139743).

### Search strategy

We systematically searched PubMed, Scopus, and the Cochrane Library from database inception until August 19, 2025, to search for relevant articles evaluating the effects of SGLT2 inhibitors on ventricular arrhythmias in patients with ICD. No restrictions were applied regarding language or publication year. We also searched the grey literature and reviewed the reference lists of relevant studies. When necessary, study authors were contacted for additional data. We performed medical subject heading (MeSH) and keyword searches using Boolean operators (AND, OR). The search terms included combinations of “SGLT2 inhibitors,” “ventricular tachycardia,” “supraventricular tachycardia,” “atrial arrhythmia,” “implantable cardioverter-defibrillator,” and “cardiovascular mortality.” All references were downloaded for consolidation, duplicate removal, and further analysis.

### Eligibility criteria

We included randomized controlled trials and observational studies enrolling adults (≥ 18 years) with an implantable cardioverter-defibrillator (ICD) who received a sodium-glucose cotransporter-2 (SGLT2) inhibitor versus placebo or usual care. Eligible studies were required to report ventricular arrhythmia, ventricular tachycardia, and supraventricular tachycardia. Secondary endpoints included non sustained ventricular tachycardia (NSVT), ICD therapies, or cardiovascular mortality.

We excluded studies enrolling pediatric/adolescent populations; reports without relevant quantitative endpoints; case reports, small uncontrolled case series, reviews, editorials, and conference abstracts without full text; and studies lacking sufficient data despite contacting the authors.

Two investigators (KM and DC) independently screened titles and abstracts for relevance. Full-text articles were assessed against inclusion and exclusion criteria. Discrepancies were resolved through discussion or adjudication by a third reviewer (MJ). Study selection was documented using a PRISMA flow diagram.

### Data extraction

Two reviewers (KM and DC) independently screened titles and abstracts, then assessed full texts to identify potentially eligible studies. The accuracy and validity of extracted data were evaluated independently, and any discrepancies were resolved by consensus. Extracted variables included the first author, publication year, country, number of participants, and baseline characteristics (age, sex, body mass index [BMI]). Outcomes included ventricular arrhythmia, ventricular tachycardia, supraventricular tachycardia, NSVT, ICD shocks, and cardiovascular mortality.

### Study outcomes

The primary outcome was total ventricular arrhythmia, ventricular tachycardia, and supraventricular tachycardia. And, the secondary endpoints included NSVT, ICD therapies, or cardiovascular mortality.

### Statistical analysis

We conducted meta-analyses using R (version 4.5.2). We pooled dichotomous outcomes and calculated risk ratios (RRs) with corresponding 95% confidence intervals (CIs) as the effect measures. Pooled analyses were performed using a random-effects model (DerSimonian and Laird method) to account for between-study variability. We assessed heterogeneity using the Chi-square test (with a significance level of α < 0.1) and quantified it using Higgins’ I² statistic, considering I² > 50% indicative of substantial heterogeneity [18]. In cases of significant heterogeneity, we performed sensitivity analyses according to leave one out method by sequentially excluding individual studies to explore potential sources.

## Results

### Study selection

**Fig. 1:**
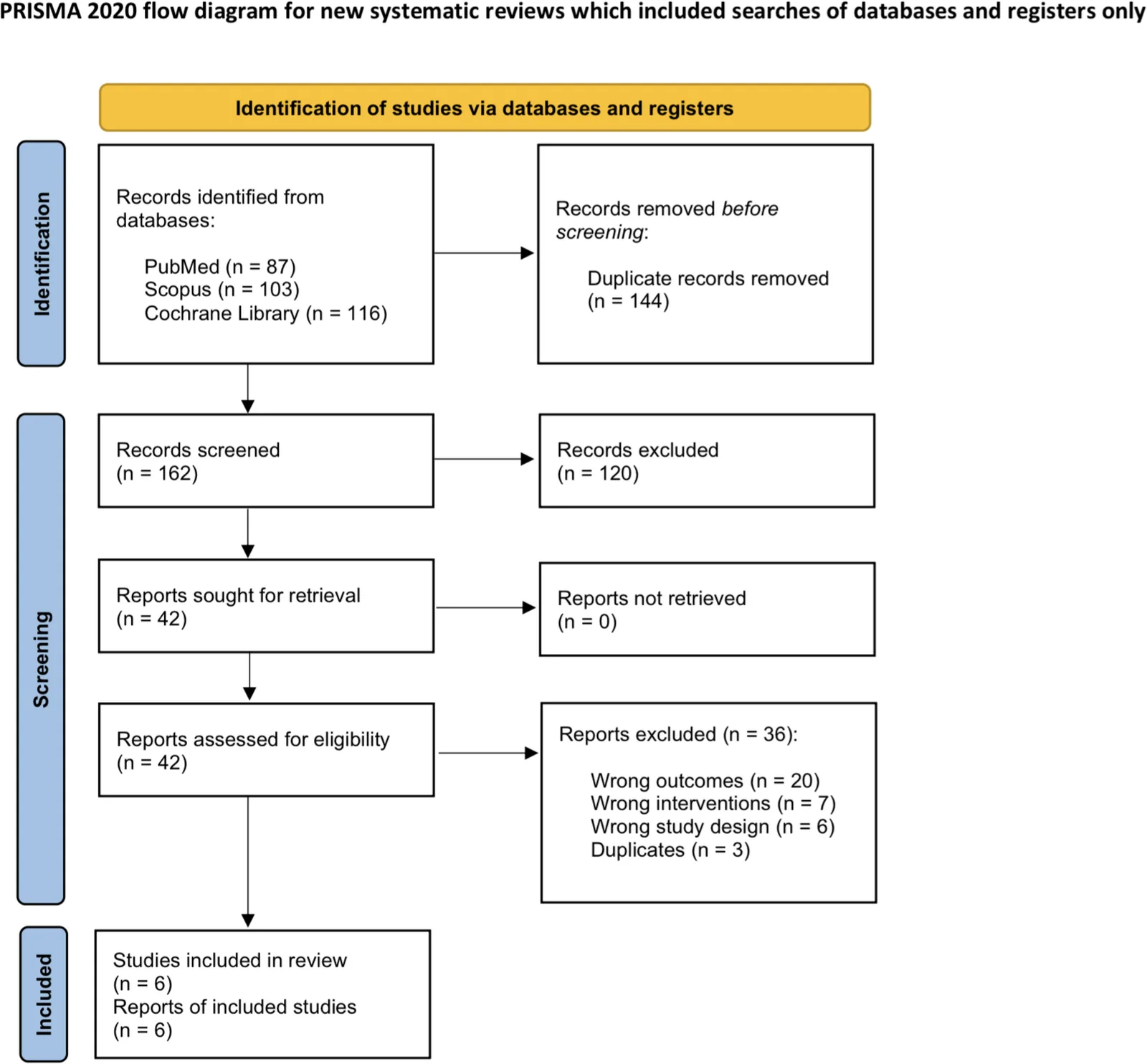
The PRISMA flowchart

The search identified 306 records (PubMed: 87; Scopus: 103; Cochrane Library: 116). After the removal of 144 duplicates, 162 unique records remained for screening. Forty-two full-text articles were assessed for eligibility, and six studies met the inclusion criteria. The reasons for the exclusion are mentioned in Fig. 1. Ultimately, six studies met the eligibility criteria and were included in this systematic review [19–24].

### Study and patient characteristics

**Fig. 2:**
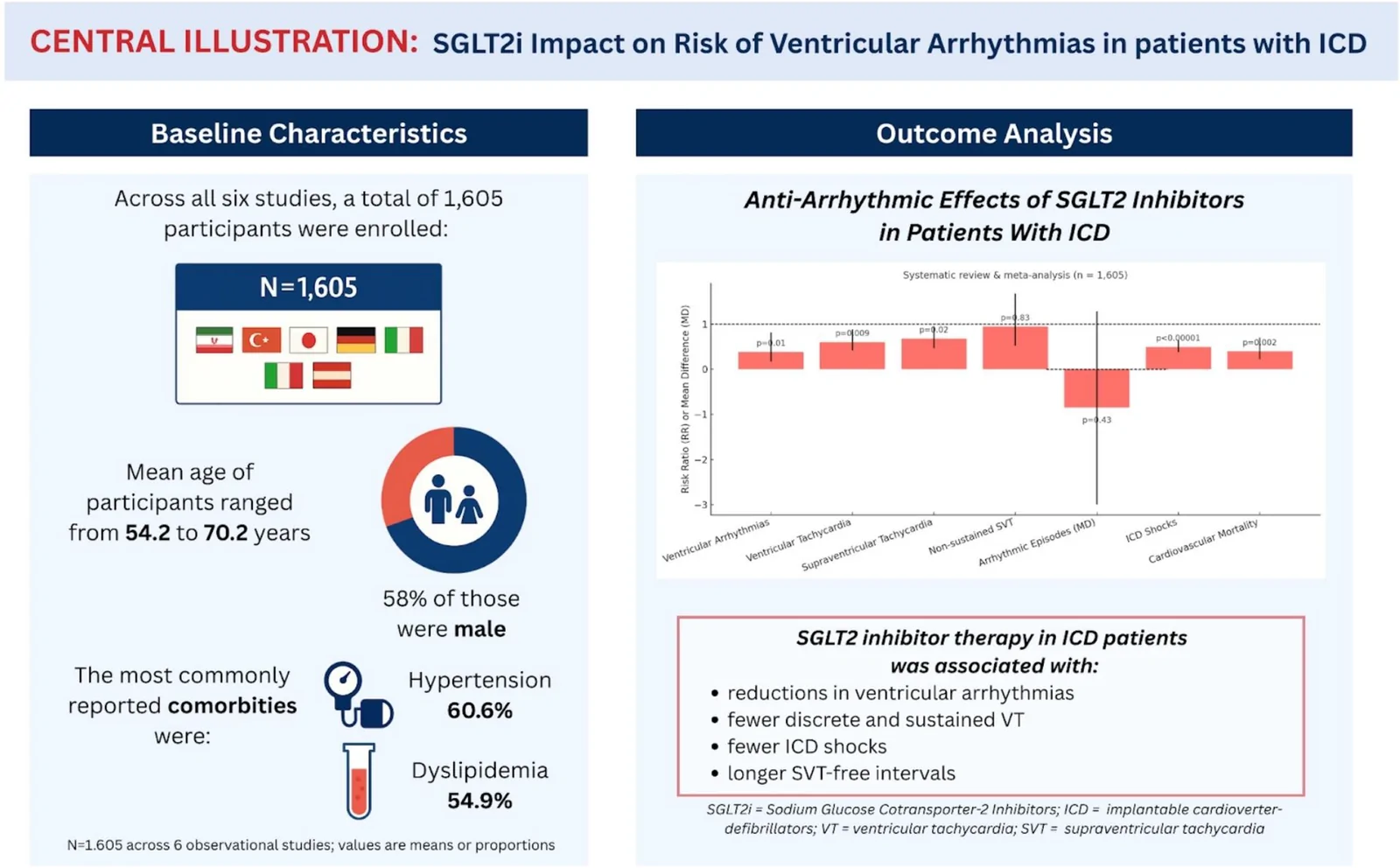
The Central Illustration

Six studies, including three randomized controlled trials and three observational studies, comprising 1,605 patients, were included in the analysis. The study characteristics and baseline patient demographics are summarized in Table 1. Overall, 941 (58.6%) of patients were male. The studies were conducted in Iran, Turkey, Japan, Germany, Italy, and Austria. Mean age ranged from 54.2 to 70.2 years. Five studies reported body mass index (BMI), with means ranging from 25.2 to 28.4 kg/m². Across the studies, dyslipidemia was present in 881 participants (54.9%), diabetes mellitus in 105 participants (6.5%), and hypertension in 973 participants (60.6%) (Fig. 2).

**Table 1:** The baseline characteristics of the included studies

Serial No.	Author	Study type	SGLT2i drug	Total Participants (*n*)	Location	Age (years)	Males (*n*)	BMI (kg/m2)	Dyslipidemia (%)	DM (%)	HTN (%)	Follow up (months)
1	Abedi et al.	RCT	Empagliflozin	SGLT2i − 35 Placebo − 37	Iran	60.36 ± 11.56	56	25.58 ± 4.82	NR	29.1	59.7	6 months
2	Erbay et al.	Observational	NR	SGLT2i − 62 Placebo − 113	Turkey	54.16 ± 14.84	103	27.64 ± 4.93	59.4	36.5	56	12 months
3	Fujiki et al.	RCT	Empagliflozin	SGLT2i − 75 Placebo − 75	Japan	70.17 ± 9.25	125	25.2 ± 4.3	56.3	NR	64	6 months
4	Katov et al.	Observational	NR	SGLT2i − 39 Placebo − 39	Germany	66.6 ± 12.9	67	28.3 ± 5.6	59	20	54	6 months
5	Sardu et al.	Observational	Empagliflozin OR Dapagliflozin	SGLT2i − 334 Placebo − 794	Italy	60.55 ± 11.31	548	28.13 ± 6.16	54.3	NR	60.4	12 months
6	Benedikt et al.	RCT	Ertugliflozin	SGLT2i − 22 Placebo − 24	Austria	65 ± 11	42	28.4 ± 5.8	NR	NR	NR	13 months

Values are reported as n (%) or mean ± standard deviation

*BMI* Body Mass Index, *NR* Not Reported

### Primary outcomes

**Fig. 3:**
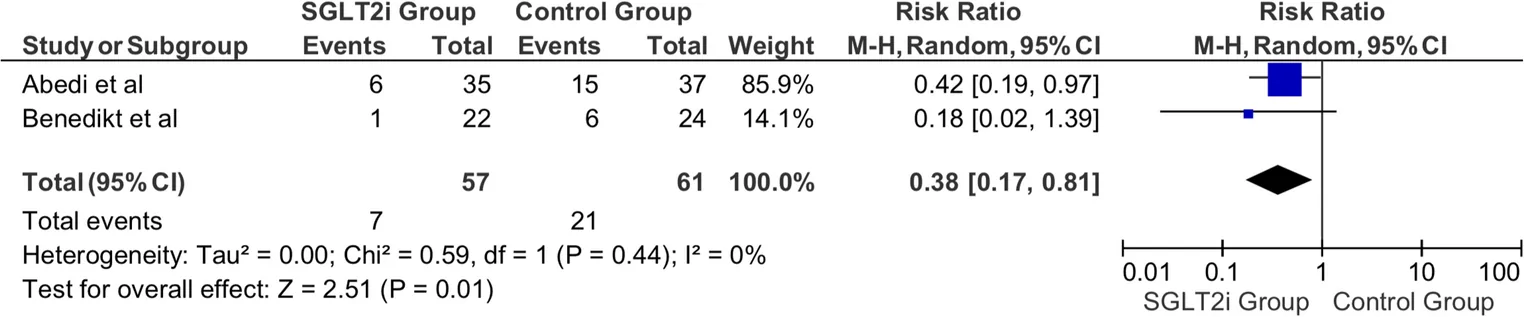
Forest plot for total ventricular arrhythmia

Our pooled analysis of total ventricular arrhythmia events in ICD patients demonstrated a significant reduction with SGLT2 inhibitor therapy. Events occurred in 7 of 57 patients (12.3%) in the SGLT2 inhibitor group, compared to 21 of 61 (34.4%) in the control group. SGLT2 inhibitors were associated with a significantly lower risk of total ventricular arrhythmia (RR: 0.38; 95% CI: 0.17 to 0.81; *p* = 0.01; I² = 0%) (Fig. 3).

**Fig. 4:**
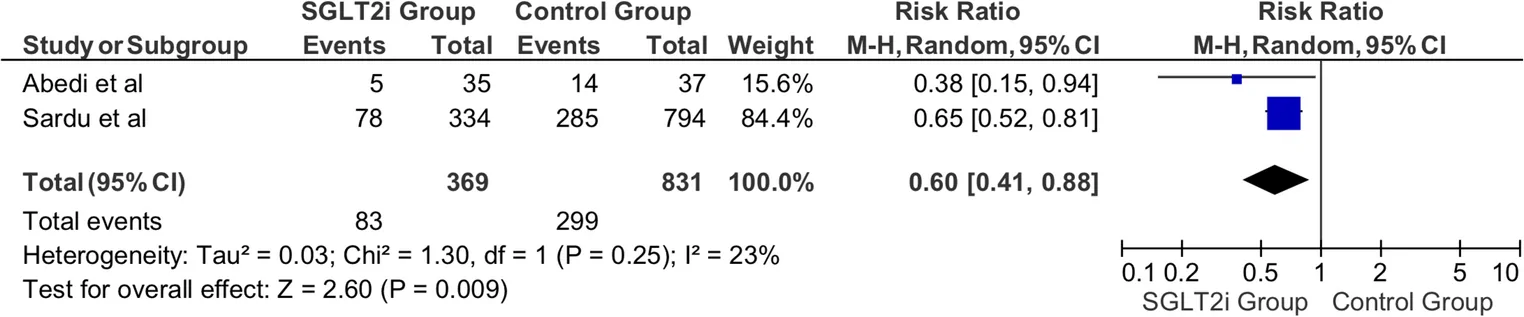
Forest plot for ventricular tachycardia events

Similarly, in two studies evaluating discrete ventricular tachycardia (VT) events, events occurred in 83 of 369 patients (22.5%) in the SGLT2 inhibitor group, compared to 299 of 831 (36.0%) in the control group. The pooled effect (RR: 0.60; 95% CI: 0.41 to 0.88; *p* = 0.009; I² = 23%) indicated fewer VT episodes among patients receiving SGLT2 inhibitors (Fig. 4).

**Fig. 5:**
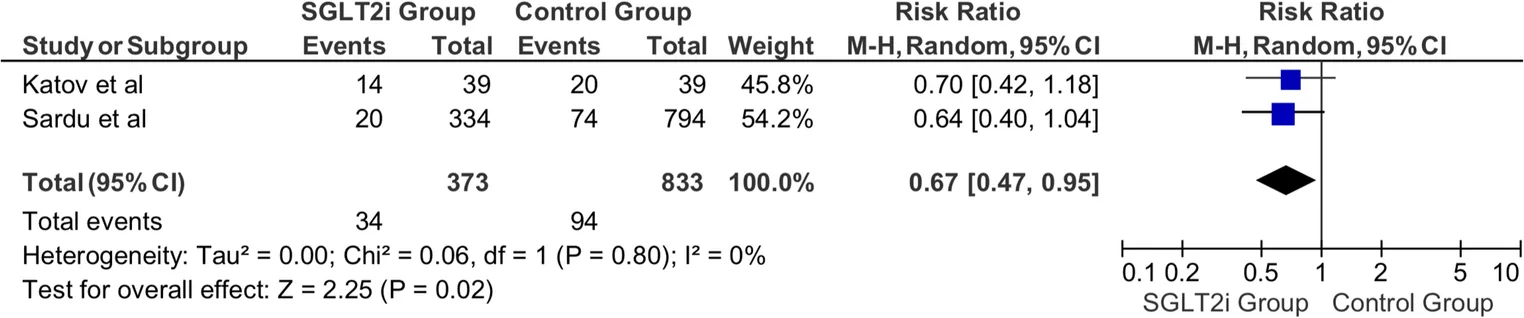
Forest plot for supraventricular tachycardia

In addition, a pooled analysis of two studies assessing supraventricular tachycardia (SVT) showed events in 34 of 373 patients (9.1%) in the SGLT2 inhibitor group, compared to 94 of 833 (11.3%) in the control group. SGLT2 inhibitor therapy was associated with a significant reduction in event rates (RR: 0.67; 95% CI: 0.47 to 0.95; *p* = 0.02; I² = 0%) (Fig. 5).

### Secondary outcomes

**Fig. 6:**
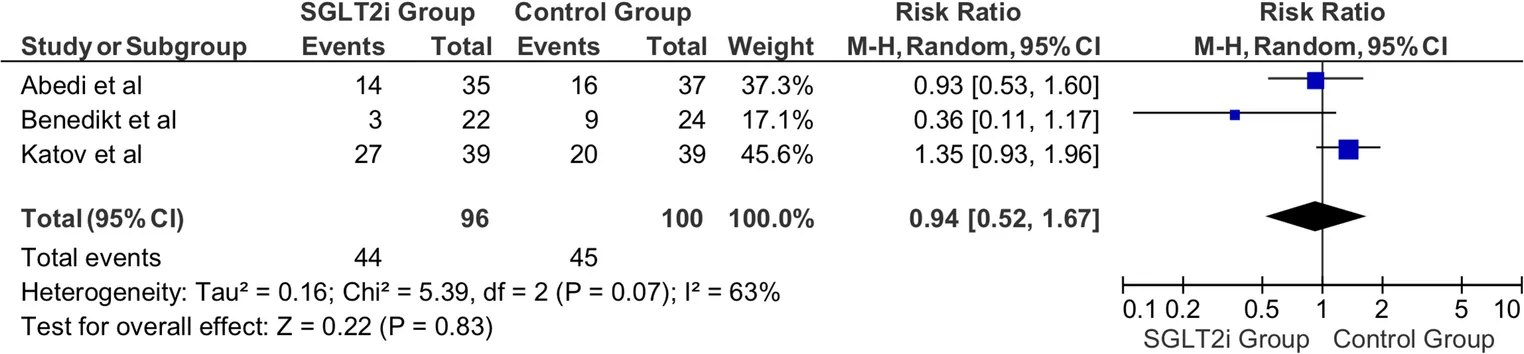
Forest plot for non-sustained ventricular tachycardia

Our pooled analysis included a total of three studies for NSVT. Events occurred in 44 of 96 patients (45.8%) in the SGLT2 inhibitor group, compared to 45 of 100 (45.0%) in the control group. The analysis found no significant difference between groups (RR: 0.94; 95% CI: 0.52 to 1.67; *p* = 0.83; I² = 63%) (Fig. 6). Sensitivity analysis following the removal of the study by Benedikt et al. showed that the heterogeneity dropped substantially from 63% to 22%; however, the risk ratio increased to 1.18 [95% CI: 0.82 to 1.69] (Supplementary Fig. 1).

**Fig. 7:**
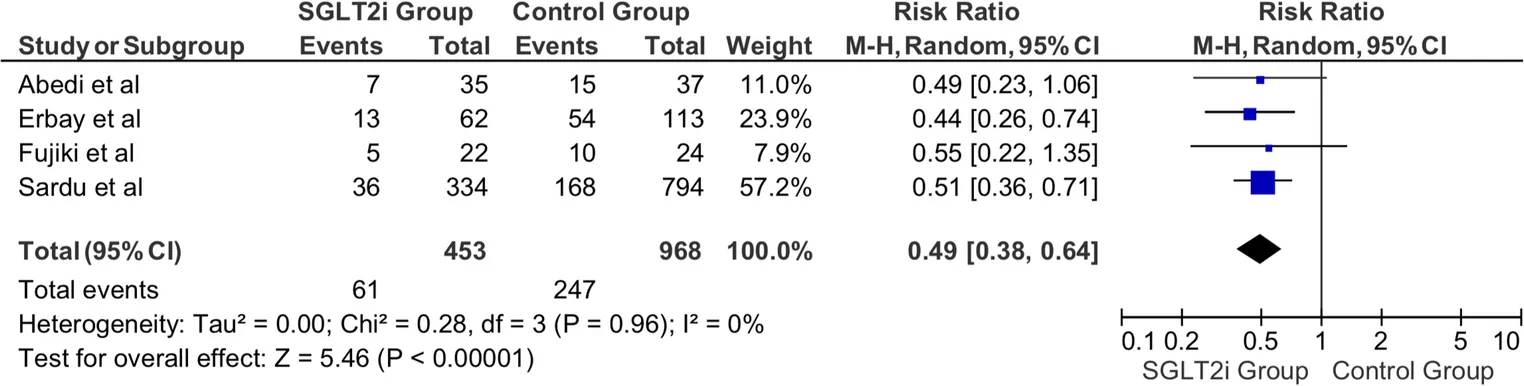
Forest plot for implantable cardioverter defibrillator shock

Across four studies evaluating ICD therapies, events occurred in 61 of 453 patients (13.5%) in the SGLT2 inhibitor group, compared to 247 of 968 (25.5%) in the control group. Pooled analysis showed a significant reduction in ICD shocks among patients treated with SGLT2 inhibitors (RR: 0.49; 95% CI: 0.38 to 0.64; *p* < 0.00001; I² = 0%) (Fig. 7).

**Fig. 8:**
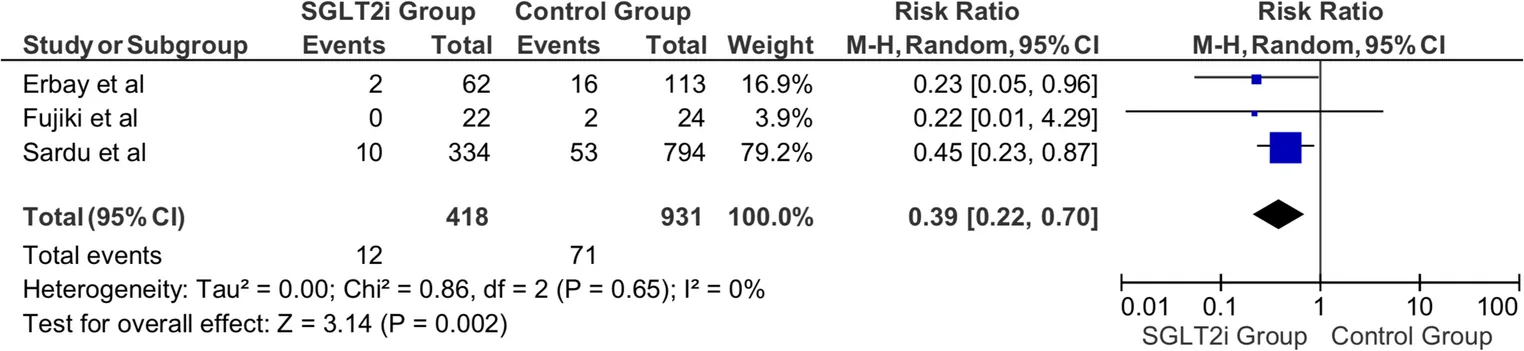
Forest plot for cardiovascular mortality

Finally, pooled data from three studies on cardiovascular mortality revealed events in 12 of 418 patients (2.9%) in the SGLT2 inhibitor group, compared to 71 of 931 (7.6%) in the control group. SGLT2 inhibitors were associated with significantly lower cardiovascular mortality (RR: 0.39; 95% CI: 0.22 to 0.70; *p* = 0.002; I² = 0%) (Fig. 8).

## Discussion

This meta-analysis demonstrated that SGLT2 inhibitor therapy in patients with ICD was associated with a significant reduction in the total ventricular arrhythmia burden and discrete ventricular tachycardia events. The use of SGLT2 inhibitors was also associated with a lower incidence of supraventricular tachycardia and a marked reduction in the need for ICD shocks. Although no significant difference was observed for NSVT, the cardiovascular mortality was reported to be significantly lower among patients receiving SGLT2 inhibitors compared with those in the control group.

Randomized trials, including MADIT-II and SCD-HeFT, established that prophylactic ICD implantation in high-risk patients reduces all-cause mortality, underscoring both the elevated baseline risk and the survival benefit conferred by ICD therapy [25, 26]. Contemporary registry data further highlight the substantial incidence of appropriate ICD therapies, particularly among secondary prevention recipients, where shocks are linked to recurrent ventricular arrhythmias and serve as a strong predictor of subsequent mortality [27, 28].

Emerging evidence suggests that SGLT2 inhibitors, when added to guideline-directed therapy, may exert beneficial effects beyond hemodynamic and metabolic improvements, potentially influencing arrhythmia risk [29]. SGLT2 inhibitor therapy has demonstrated a consistent signal toward reduction in ventricular arrhythmic burden among patients with heart failure and or type 2 diabetes who had ICD or CRT-D devices. Sardu et al. showed a significant reduction in both the frequency and proportion of VT/VF episodes, with a parallel trend toward fewer ICD shocks, particularly in patients already receiving antiarrhythmic drugs [23]. Similarly, the EMPA-ICD trial reported fewer ventricular arrhythmias with empagliflozin compared with placebo in ICD or CRT-D recipients with type 2 diabetes [21]. The study by Benedikt et al. also observed fewer sustained VT or VF events with SGLT2 inhibition versus placebo [24]. Prior meta-analyses have reported variable results regarding arrhythmic outcomes, with the effect on ventricular arrhythmias remaining uncertain. Jaiswal et al. similarly found reduced rates of atrial arrhythmias and SCD in patients with diabetes, CKD, and HFrEF treated with SGLT2 inhibitors. However, they observed no significant reduction in ventricular arrhythmias [30]. Importantly, high-risk populations with ICDs have generally been underrepresented in these studies. Our meta-analysis specifically focused on this cohort, leveraging intracardiac ICD recordings to provide a precise assessment of ventricular arrhythmia burden and device therapies.

Our analysis showed that SGLT2 inhibitor therapy was associated with a lower composite burden of ventricular arrhythmias compared with placebo. Similarly, patients receiving SGLT2 inhibitors experienced fewer ventricular tachycardia events. These findings are consistent with the meta-analysis by Lin et al., which reported reduced ventricular arrhythmia and sudden cardiac death among patients with HF irrespective of their diabetes status [15]. In contrast, meta-analyses by Wang et al. and Liao et al. did not show a similar effect and enrolled studies with a broader population such as individuals with HF, chronic kidney disease and/or type 2 diabetes [12, 13]. These divergent results likely reflect differences in baseline risk and outcome ascertainment: our population included ICD recipients, a group with higher arrhythmic susceptibility and continuous device-based monitoring, whereas prior meta-analyses included lower-risk populations in whom event rates and duration of follow-up may have been insufficient to detect benefit. Collectively, these observations suggest that any antiarrhythmic effect of SGLT2 inhibitors is most apparent in higher-risk populations and when ventricular arrhythmias are rigorously captured by device diagnostics.

SGLT2 inhibitors may lessen ventricular arrhythmia burden through complementary hemodynamic, autonomic, and cellular mechanisms. Hemodynamic unloading from natriuresis and osmotic diuresis reduces filling pressures and ventricular wall stress, favoring electrical stability [9, 10, 14]. In parallel, anti-inflammatory and anti-fibrotic remodeling plausibly decreases the risk of arrhythmia. Recent evidence in pre-clinical experimental studies suggests that Empagliflozin treatment leads to fibrosis regression and improves myocardial structure, reducing fixed re-entry circuits and dispersion of conduction [31]. Autonomic modulation has also been observed in similar pre-clinical trials, with reductions in sympathetic nerve activity and improved baroreflex control [32]. At the cellular level, empagliflozin can inhibit the cardiac Na⁺/H⁺ exchanger (NHE1), lowering intracellular Na⁺ and secondary Ca²⁺ overload, thereby limiting delayed afterdepolarizations and triggered ventricular tachycardia [31, 33]. Collectively, these effects align with the observed reductions of ventricular arrhythmias as noted by our analysis.

In our secondary analysis, SGLT2 inhibitor therapy did not reduce the burden of NSVT compared with placebo across the studies that reported this endpoint [19, 22]. Interpretation is limited, as only two studies contributed to NSVT data, leading to imprecision and restricted generalizability. Variation in device programming threshold and in arrhythmia detection likely contributed to variability in effect estimates. Differences in the distribution of ischemic versus nonischemic cardiomyopathy may also have confounded results, as noted in Katov et al. [22]. These findings suggest that SGLT2 inhibitors may exert a more consistent anti-arrhythmic effect on sustained ventricular arrhythmias. Confirmation will require larger studies with standardized device settings, adequate follow-up, and balanced populations.

When compared with placebo, SGLT2 inhibitor therapy was associated with a higher likelihood of remaining free from supraventricular tachycardia (SVT). This finding aligns with prior meta-analyses demonstrating lower rates of atrial tachyarrhythmias-particularly reductions in atrial fibrillation and atrial flutter burden [12, 13]. The treatment with SGLT2 inhibitors was also associated with lower cardiovascular mortality compared with placebo. This observation is consistent with the attenuation of malignant ventricular arrhythmias identified in our primary analysis and reflects the broader cardioprotective profile of SGLT2 inhibitors demonstrated in prior large-scale HF trials and meta-analyses [9–11, 34, 35].

Furthermore, SGLT2 inhibitor therapy was associated with fewer ICD shocks than placebo, suggesting suppression of sustained ventricular arrhythmias that typically trigger device interventions. Given the established associations between ICD shocks and worse quality of life, rehospitalization, and mortality, this finding carries notable clinical significance [5, 36]. Despite variations in device programming and baseline risk across studies, the consistent direction of benefit and concordant reduction in ventricular arrhythmia burden support the role of SGLT2 inhibitors as a potential adjunctive therapy in ICD recipients.

### Limitations

Several limitations should be considered. There was notable heterogeneity across the included studies in terms of design, eligibility criteria, baseline risk profiles, and endpoint definitions. This variability may have influenced the observed outcomes and limits the comparability of results across studies. The differences in device programming across studies may have introduced variability in the incidence of therapies, potentially affecting the pooled estimates. The follow-up duration was relatively short in several studies, which could have led to underestimation of benefits that may become more apparent over longer periods.

Our analysis included both randomized controlled trials (RCTs) and observational studies. While appropriate statistical pooling methods were used, the inclusion of observational studies inherently increases the risk of bias due to confounding and selection factors. Notably, a single large observational study contributed over half of the total patient population, which may have disproportionately influenced the overall pooled estimates, introducing weighting bias and potentially limiting the robustness of the conclusions.

Additionally, our analysis relied on published study-level data rather than individual patient-level data. This prevented adjustment for patient-level covariates, restricted analysis of time-to-event outcomes, and limited the ability to apply standardized adjudication of appropriate versus inappropriate therapies. Although an RCT-only sub-analysis would have provided more rigorous evidence, it could not be conducted due to insufficient available data.

Finally, these limitations collectively highlight the need for adequately powered, large-scale randomized trials in implantable cardioverter-defibrillator (ICD) recipients, with standardized device programming and consistent endpoint definitions, to more definitively evaluate the outcomes of interest.

## Conclusion

SGLT2 inhibitors were associated with a lower risk of overall ventricular arrhythmias, ventricular tachycardia, SVT, ICD interventions, and cardiovascular mortality in patients with ICDs. These associations suggest that SGLT2 inhibitors may have potential protective effect on arrhythmic burden and cardiovascular outcomes in this high-risk population, which could be mediated through their favorable effects over cardiac remodeling, electrophysiologic stability, and the overall myocardial function. These findings support further investigation of SGLT2 inhibitors as adjunctive therapy in ICD patients receiving guideline-directed care. However, confirmation in large-scale, randomized controlled trials is needed to definitively establish their efficacy and long-term safety for arrhythmic outcomes in this population.

## Supplementary Information

Below is the link to the electronic supplementary material.

Supplementary Material 1 (DOCX 170 KB)

## Data Availability

No datasets were generated or analysed during the current study.

## Declarations

### Approval of the research protocol

No human participants were involved in this study and no new data was synthesized.

### Animal Studies

N/A.
