# Supplementary material for: The impact of sodium glucose cotransporter-2 inhibitors on ventricular arrhythmia in patients with implantable cardioverter-defibrillators: a meta analysis
Source: J Interv Card Electrophysiol. 2026 Feb 28;69(7):2031–9. doi: 10.1007/s10840-026-02274-1 (PMC13687273; doi:10.1007/s10840-026-02274-1)
Supplement: Supplementary file 1 [file 10840_2026_2274_MOESM1_ESM.docx]

**Legend:**

**Supplementary Figure 1:** Forest plot of leave-one-out analysis for non-sustained ventricular tachycardia

**Supplementary Figure 1:** Forest plot for leave-one-out analysis for non-sustained ventricular tachycardia
